# Postoperative Complications and Stoma Rates After Laparoscopic Resection of Deep Infiltrating Endometriosis with Bowel Involvement

**DOI:** 10.1055/s-0042-1756212

**Published:** 2022-09-22

**Authors:** Rogério Serafim Parra, Fernando Passador Valério, José Vitor Cabral Zanardi, Marley Ribeiro Feitosa, Hugo Parra Camargo, Omar Féres

**Affiliations:** 1 School of Medicine of Ribeirão Preto, Universidade de São Paulo, Ribeirão Preto, SP, Brazil

**Keywords:** colorectal surgery, endometriosis, laparoscopy, postoperative complications, surgical stomas, cirurgia colorretal, endometriose, laparoscopia, complicações pós-operatórias, estomas cirúrgicos

## Abstract

**Objective** The purpose was to assess the rates of postoperative complications and the need of temporary stoma of laparoscopic surgical treatment for bowel endometriosis in a referral center.

**Methods** The surgical indication, type of operation, operative time, length of hospital stay, need for a temporary stoma, rate of conversion to open surgery, postoperative complications were evaluated.

**Results** One-hundred and fifty patients were included. The average duration of surgery was significantly longer for segmental resection (151 minutes) than for disc excision (111.5 minutes, p < 0.001) and shaving (96.8 minutes, p < 0.001). Patients with segmental resection had longer postoperative lengths of hospital stay (1.87 days) compared with patients with disc excision (1.43 days, p < 0.001) and shaving (1.03 days, p < 0.001). A temporary stoma was performed in 2.7% of patients. Grade II and III postoperative complications occurred in 6.7% and 4.7% patients, respectively.

**Conclusion** Laparoscopic intestinal resection has an acceptable postoperative complication rate and a low need for a temporary stoma.

## Resumo

**Objetivo** O objetivo foi avaliar as taxas de complicações pós-operatórias e a necessidade de estomia temporária do tratamento cirúrgico laparoscópico para endometriose intestinal em um centro de referência.

**Métodos** Foram avaliados a indicação cirúrgica, tipo de operação, tempo operatório, tempo de internação, necessidade de estomia temporária, taxa de conversão para cirurgia aberta, complicações pós-operatórias.

**Resultados** Cento e cinquenta pacientes foram incluídos. A duração média da cirurgia foi significativamente maior para a ressecção segmentar (151 minutos) do que para a excisão do disco (111,5 minutos, *p* < 0,001) e *shaving* (96,8 minutos, *p* < 0,001). Pacientes com ressecção segmentar tiveram maior tempo de internação pós-operatória (1,87 dias) em comparação com pacientes com excisão de disco (1,43 dias, *p* < 0,001) e shaving (1,03 dias, *p* < 0,001). Um estoma temporário foi realizado em 2,7% dos pacientes. Complicações pós-operatórias de grau II e III ocorreram em 6,7% e 4,7% dos pacientes, respectivamente.

**Conclusão** A ressecção intestinal laparoscópica apresenta taxa aceitável de complicações pós-operatórias e baixa necessidade de estomia temporária.

## Introduction

Endometriosis is a chronic inflammatory disease with a high prevalence among women of reproductive age. 1 Deep infiltrating endometriosis (DIE) is one of the most severe types of endometriosis, and in up to 25% of cases, it affects the bowel. 2 Lesions can be single or multifocal, and depending on the anatomical site affected, they can present symptoms that vary from dysmenorrhea to dyspareunia, and rectal bleeding. 3

Preoperative assessment is crucial to determine the best therapeutic approach for intestinal DIE. 4 This planning consists of clinical examinations and imaging, to identify the location, such as the rectum, vagina, uterosacral ligaments, bladder, and ureter. Imaging can predict the presence of DIE and define the size and extent of lesions compromising the bowel. 5 Lesions affecting the ileum, cecum, or the appendix are unusual and can be difficult to identify with preoperative imaging. Therefore, careful and methodical inspection of these structures must be performed during laparoscopy. 6

Patient age, pain intensity, risk of intestinal obstruction and desire for pregnancy are factors that should be considered for the management of DIE with bowel involvement. Surgery is mainly indicated in patients with pelvic pain who do not respond to medical therapy and in those with the desire for pregnancy. 2 6 Laparoscopic segmental resection, rectal disc excision, and rectal shaving are the main surgical techniques described to treat DIE infiltrating the intestine. The coexistence of two or more lesions affecting the bowel requires segmental resection of the bowel. 5 Rectal disc excision should be reserved for patients with single lesions smaller than 3 cm. Ileal disease also requires surgical excision because of the high risk of acute obstruction. 7 When the appendix is affected, surgery is always indicated to rule out carcinoid tumors. 8 Surgical removal of the lesion is also required when lesions are symptomatic, impairing bowel, urinary, sexual, and reproductive functions. Complete resection should be attempted to reduce the risk of residual lesions and recurrence. 6 9

Postoperative complications represent one of the greatest concerns for bowel surgeries, including leakage, fistulas, and severe peritonitis. 10 Another fear is whether the patients will need a temporary stoma. The aim of the present study was to assess the rates of postoperative complications and the need for temporary stoma in laparoscopic surgical treatment for bowel endometriosis at a single referral center and to compare them with the outcomes in the available literature.

## Methods

We conducted a retrospective study using electronic data prospectively recorded. We included all patients with DIE with bowel involvement submitted to laparoscopy with intestinal resection from October 2014 to October 2019. Patients submitted to open surgery as the main surgery or those without bowel involvement were excluded. We evaluated the following patient's characteristics: age, body mass index (BMI), previous surgeries, surgical indication, type of operation, operative time, length of hospital stay, need for temporary stoma, conversion rate to open surgery, and need for a second bowel resection. We also evaluated grades II and III postoperative complications according to the Clavien-Dindo classification. 11

All surgical procedures were performed by the same surgical team, composed by a colorectal surgeon and three gynecologists. Surgery was mainly indicated in patients with pelvic pain who did not respond to medical therapy as well as those with desire for pregnancy. We also indicated surgery in ileal disease because of the high risk of bowel obstruction, and when the appendix was affected, to rule out carcinoid tumors. The criteria to perform either rectal shaving, disc excision or segmental resection depended on location, number, size, and circumferential involvement of the lesions as described elsewhere. 2 In summary, lesions larger than 3 cm in diameter, multifocal bowel lesions or extent of bowel circumference involvement higher than 40% required a segmental resection. Disc excision was the preferred technique in the presence of unique nodules, lower than 3 cm in diameter and with lower than 40% of circumference involvement. Rectal shaving was planned to be performed in patients without involvement of inner layer muscularis or deeper. Some patients required more than one bowel resection for DIE. When two or more surgical procedures for DIE were performed on the same patient, we considered the major surgical procedure in the statistical analysis, to assess the outcomes.

Our imaging protocol includes evaluation with transvaginal ultrasound with a high-resolution linear transducer and bowel preparation in all patients to map the endometriosis lesions of the right iliac fossa and to detect lesions of the ileum, cecum, and appendix. Previous studies showed that ultrasonography has diagnostic accuracy not inferior to magnetic resonance imaging. 12 13 The diagnostic performance of transvaginal ultrasound and magnetic resonance imaging are similar for detecting deep endometriosis involving the rectosigmoid colon, uterosacral ligaments, and rectovaginal septum. Therefore, it must be considered the primary approach for deep endometriosis diagnosis.

Categorical variables were expressed as frequencies/percentages and continuous variables as means ± standard deviations. The one-sample Kolmogorov-Smirnov test was used to assess the normality of continuous variables. Analysis of variance was used to compare continuous variables. Fisher exact test or the χ2 test was used to compare categorical variables. All *p* -values were 2-sided, and a significance level of 5% was established. Statistical analysis was performed with IBM SPSS Statistics for Windows, Version 20.0 (IBM Corp., Armonk, NY, USA).

The study was approved by the ethics committee of the Hospital das Clínicas of Faculdade de Medicina de Ribeirão Preto, Universidade de São Paulo (HCFMRP-USP) (IRB protocol, CAAE: 31679420.3.0000.5440; Ethics Committee Approval: 4.029.839/2020). All procedures were conducted in accordance with the 1964 Declaration of Helsinki and its later amendments or comparable ethical standards.

## Results

### Demographic Characteristics

A total of 150 women with DIE and bowel involvement who underwent laparoscopic surgical management were included. The mean age was 35.1 ± 5.3 years, and the mean body mass index (BMI) was 25.1 ± 3.8 kg/m ^2^ . Most patients were nullipara (n = 111; 74%), and in 38.7% (n = 58), it was the first attempt at resection of DIE. Ninety-two patients (61.3%) had already been previously subjected to surgical management for endometriosis, and, of these, 7 patients had undergone recent surgical treatment (less than 1 year) in another hospital and were considered to have “frozen pelvis”. The demographic data are shown in Table 1 .

**Table 1:** Demographic data (n = 150)

Patients' characteristics	Observed
Age (mean ± SD) (min–max) (years)	35.1 ± 5.3 (20–53)
BMI (mean ± SD) (kg/m ^2^ )	25.1 ± 3.8
Previous surgeries for endometriosis n (%)	92(61.3)
Previous pregnancies n (%) Nulliparous Previous birth	111 (74) 39 (26)

Abbreviations: BMI, body mass index; SD, standard deviation

### Surgical Indication and Surgical Procedures

The main surgical indication was severe chronic pelvic pain refractory to medical management (66%), followed by infertility (56%), dyspareunia (46%), and dysmenorrhea (34.7%). The rectosigmoid was the most frequently affected organ of the gastrointestinal tract (98.7%). The appendix was involved in 10.7% (n = 16) of cases. The analysis of the appendix of one patient revealed a well-differentiated neuroendocrine carcinoma at the appendix tip. 14 The ileum was involved in 6% of cases. Only 2 patients (1.3%) did not have rectosigmoid involvement and underwent laparoscopy: one due to isolated small bowel (ileal) involvement and the other due to ileal and appendix involvement. These results are shown in Table 2 .

**Table 2:** Surgical indication and bowel involved in the patients with DIE

Surgical indication	N (%)
Chronic pelvic pain Infertility Dyspareunia Dysmenorrhea	99 (66) 84 (56) 69 (46) 52 (34.7)
**Bowel involved**	**N (%)**
Rectosigmoid Appendix Small bowel Cecum	148 (98.7) 16 (10.7) 5 (3.3) 4 (2.7)

Abbreviations: DIE, deep infiltrating endometriosis

Eighty-three percent of patients (n = 124) had uterosacral ligament or retrocervical space involved. An endometrioma was present in 70.6% (n = 106) of the patients, and adenomyosis was present in 35.3% of the cases (n = 53). The median size of the rectosigmoid nodules was 2.1 cm (range 0.8–6.5 cm). In 69.6% of the patients (n = 103), there was a single rectal nodule, and in 47 patients (31.7%), there were 2 of more rectosigmoid nodules. In 41 patients, the rectosigmoid nodule was located up to 8 cm from the anal verge (27.7%), and in 103 patients (69.6%), the lesion was located 9 to 15 cm from the anal verge. In 6 cases (4.1%), the lesions were located more than 15 cm from the anal verge. Segmental resection was performed in 73 patients (48.7%). Rectal disc excision and shaving for bowel endometriosis were performed in 45 (30.0%) and 35 (23.3%) patients, respectively. The associated surgical procedures performed were hysterectomy (n = 19; 12.7%), appendectomy (n = 16; 10.7%), ureter nodule excision (n = 8; 5.3%), and ileocolic and/or small bowel resection (n = 9; 6%). Hysterectomies were performed in 19 patients due to concomitant endometriosis and either adenomyosis or leiomyomatosis; these patients had previously failed clinical treatment and had no future reproductive desire. Bowel endometriosis was histologically confirmed in all patients. These results are shown in Table 3 .

**Table 3:** Type of operations

Type of operations	N (%)
Shaving Shaving (only) Shaving + Hysterectomy Shaving + rectal disc excision Shaving + appendectomy Shaving + ureter nodule excision Shaving + segmental resection + appendectomy Rectal disc excision Rectal disc excision (only) Rectal disc excision + hysterectomy Rectal disc excision + appendectomy Rectal disc excision + shaving Rectal disc excision + segmental resection Rectal disc excision + ureter nodule excision Rectal disc excision + ileal resection + appendectomy Segmental resection (rectosigmoid) Segmental resection (only) Segmental resection + Hysterectomy Segmental resection + ureter/bladder nodule excision Segmental resection + appendectomy Segmental resection + ileal resection + appendectomy Segmental resection + ileal resection Segmental resection + ileocecal resection Segmental resection + appendectomy + ureter nodule excision Segmental resection + shaving + appendectomy Segmental resection + rectal disc excision Ileal resection (without rectal involvement)	35 (24.0) 21 (14.0) 5 (3.3) 3 (2.0) 3 (2.0) 2 (1.3) 1 (0.67) 45 (30) 29 (19.3) 6 (4.0) 4 (2.7) 3 (2.0) 1 (0.67) 1 (0.67) 1 (0.67) 73 (48.7) 49 (32.7) 8 (5.3) 4 (2.7) 3 (2.0) 2 (1.3) 2 (1.3) 2 (1.3) 1 (0.67) 1 (0.67) 1 (0.67) 2 (1.3)

**Fig. 1:**
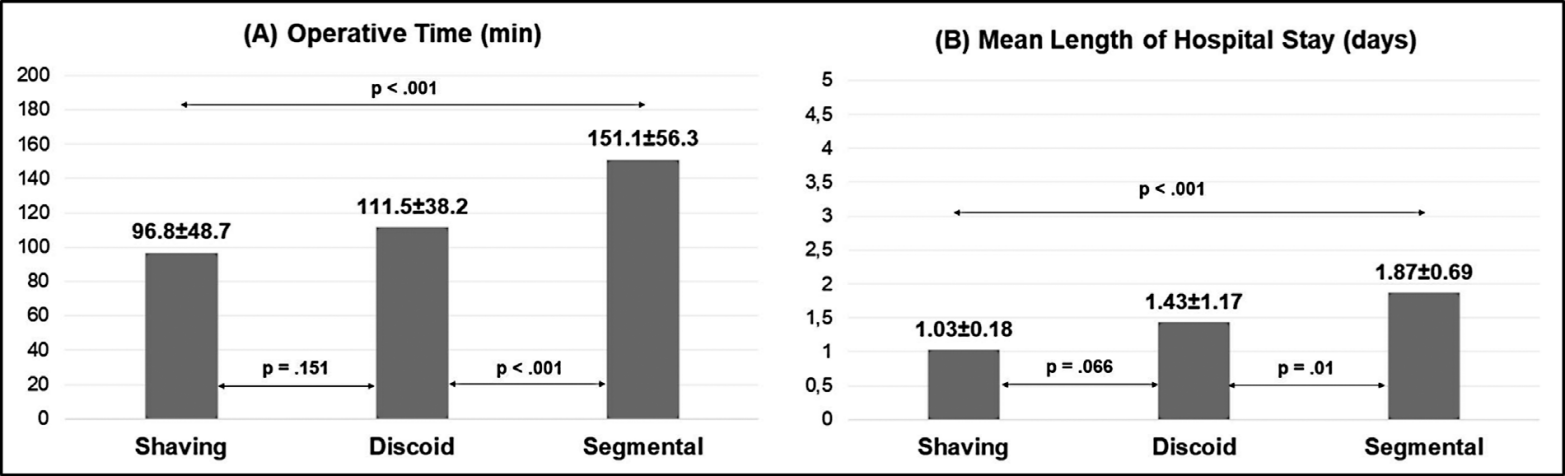
Comparative analysis of operative time (A), mean length of hospital stay (B). Statistically significant differences between groups were demonstrated by arrows with the corresponding *p* -value.

The average operative time was 128 ± 55 minutes. The mean duration of the procedure was longer for segmental resection (151 ± 56.3 minutes) than for disc excision (111.5 ± 38.2 minutes, *p* < 0.001) and shaving (96.8 ± 48.7 minutes, *p* < 0.001). Patients with segmental resection had longer postoperative lengths of hospital stay (1.87 ± 0.69 days) than those with disc excision (1.43 ± 1.17 days, *p* < 0.001) and shaving (1.03 ± 0.18 days, *p* < 0.001). These results are shown in Figure 1 .

### Postoperative Complications

Grade-II complications occurred in 6.7% of patients (n = 10). One patient who underwent disc excision had excessive rectal bleeding within 24 hours of the postoperative period and required transfusion without the need for endoscopic hemostasis. There were five patients with surgical site infections, all of whom were successfully treated clinically, and there was one patient with deep vein thrombosis. Two patients had diarrhea within 30 days postoperatively and required hospitalization and intravenous antibiotics. One patient who underwent disc excision required bladder self-catheterization and urinary physiotherapy for 6 months due to bladder atony. Upon clinical follow-up, there was complete resolution of symptoms.

Grade-III complications requiring surgical intervention occurred in 4.7% of patients (n = 7). Two patients had thermal injuries of the rectum during laparoscopy, and both cases were detected within 36 hours of the 1 ^st^ surgery. In the patient who underwent disc excision, the abdominal cavity was cleaned, and sigmoid ostomy was performed. In the patient undergoing shaving, there was a need for segmental resection of the rectum, with anastomosis and protective ostomy. One patient developed a pelvic abscess, with the need for another laparoscopy 47 days after the 1 ^st^ surgery. In three patients, there was an accidental injury to the ureter detected during laparoscopy. Last, 1 patient presented with a urinoma, and a late thermal bladder injury was detected on the 17 ^th^ postoperative day requiring a laparoscopic surgical re-approach and ureter reimplantation. Intensive care was not required in any of the patients. The major postoperative surgical complications (grade III) are presented in Table 4 .

**Table 4:** Major complications, classified as grade III of the Clavien-Dindo classification (n = 7)

Complication	N (%)
Urinary complications	4 (2.7)
Shaving Disc excision Segmental resection Infected hematoma of the Douglas cul-de-sac Shaving Disc excision Segmental resection	0 (0) 0 (0) 4 (2.7) 1 (0.67) 0 (0) 0 (0) 1 (0.67)
Thermal bowel injury Shaving Disc excision Segmental resection	2 (1.3) 1 (0.67) 1 (0.67) 0 (0)

### Conversion Rates, and the Need for Temporary Stoma and fora Second Bowel Resection

The rate of conversion to open surgery was 2.0% (n = 3). A temporary stoma was performed in 4 patients (2.7%), and this outcome was not different among the 3 surgeries. In 2 patients, the indication for the stoma was due to thermal injury to the intestine; one protective stoma was performed in a patient who underwent rectal disc excision with leakage detected intraoperatively, and another protective stoma was performed in a patient with very low (< 3 cm from the anal verge) colorectal anastomoses. The need for a 2 ^nd^ laparoscopy for the resection of intestinal nodules occurred in 5 patients during an average follow-up time of 28.9 ± 16.7 months. All of these patients had postoperative image evaluation showing no residual lesions in the intestine after the first surgery. There were no cases of anastomosis dehiscence or death. These results are summarized in Table 5 .

**Table 5:** Short-term postoperative outcomes

Outcome	N (%)
Conversion rate to open surgery Shaving Disc excision Segmental resection	3 (2.0) 0 (0) 1 (0.67) 2 (1.3)
Need of temporary stoma Shaving Disc excision Segmental resection	4 (2.7) 1 (0.67) 2 (1.3) 1 (0.67)
30-day emergency room visit Shaving Disc excision Segmental resection	5 (3.3) 1 (0.67) 0 (0) 4 (2.7)
30-day reoperation rate Shaving Disc excision Segmental resection	3 (2.0) 1 (0.67) 1 (0.67) 1 (0.67)
Surgical recurrence Shaving Disc excision Segmental resection Blood transfusion Shaving Disc excision Segmental resection Bladder atony Shaving Disc excision Segmental resection Dehiscence of intestinal anastomosis Overall mortality	5 (3.3) 2 (1.3) 2 (1.3) 1 (0.67) 1 (0.67) 0 (0) 1 (0.67) 0 (0) 1 (0.67) 0 (0) 1 (0.67) 0 (0) 0 (0) 0 (0)

## Discussion

Our study showed that laparoscopic surgery for the treatment of DIE with bowel involvement was safe, with very low conversion rates to open surgery and without the routine use of stoma during the initial surgery. This did not negatively impact the results of our cohort of patients, who had low rates of serious postoperative complications and low need for second laparoscopy for the resection of intestinal nodules due to recurrence.

Among all the patients in the study, four needed a temporary stoma. All stomas were subsequently reversed within a period of up to 3 months after the first surgery. The stoma rate was lower in our study than the average rate reported in the literature. Ruffo et al. 15 reported that 21.3% of patients required an ileostomy, and stoma was still present at the time of follow-up in 3.2% of patients (5 of 156 stomas). Another study compared conservative surgery (rectal shaving or disc excision) versus segmental resection and showed that 59.3% of patients who underwent conservative surgery needed a colostomy and 71.4% of patients who underwent segmental resection needed a colostomy (42.9%) or ileostomy (28.6%). 16 A protective stoma was performed in 27% of patients in a prospective cohort study, 17 and in a case-series study using mainly conventional laparoscopy, the stoma rate was 19.1%. 18

Our study showed no differences in the stoma rate among the three surgeries, even if we compared conservative surgery with segmental resection. The indication for a protective stoma should be individualized, depending on risk factors for leakage, and should be recommended only for a low coloanal anastomosis 3 or when the anastomosis is performed lower than 5 cm from the anal verge due to the risk of anastomotic leakage. 19 20 Laparoscopic surgery for DIE with bowel involvement is almost always elective and scheduled, mostly in young patients: those without comorbidities (ASA 1 or 2), those with no prolonged operative time (< 3 hours), and those with no anemia, malnutrition, or any other factors that could increase the risk of dehiscence, such as anemia or sepsis. 21 In our series, there were no cases of anastomotic leakage, which is very similar to other series that showed 0.8% of cases of anastomotic leakage. 18

A stoma may be associated with postoperative complications, such as abnormal healing of the stoma scar, wound or urinary infections following stoma closure, leakage, hernias, subcutaneous abscesses, and bowel obstruction. 22 Additionally, those patients will require another surgical intervention.

Serious complications occurred in 7 patients and included thermal damage to the intestine and urinary system, damage to the ureter, and postoperative infection requiring hospitalization. Abo et al. 23 showed 11.8% of patients with Clavien 3b postoperative complications, two thirds of whom were managed by segmental colorectal resection. They also reported 14 cases (3.8%) of rectovaginal fistula and 24 cases (6.6%) of pelvic abscess. Malzoni et al. 24 described a rate of 8.06% of major postoperative complications. Tarjanne et al. 25 found that the rate of major complications was 12%, and a rectal nodule higher than 4 cm was associated with the development of a major complication. There are some frequently observed risk factors of major complications. One of them is the excessive use of electrocoagulation, which increases the risk of rectovaginal fistulae and abscesses due to the risk of necrosis of the posterior vaginal cuff. In all surgeries in our cohort, we used an ultrasonic energy device, and laparoscopic bipolar electrosurgical devices were used, which may be associated with lower postoperative complications.

In one patient, a ureteral fistula was diagnosed, attributed to thermal injury. This is in accordance with a large series that reported an incidence of ureteral fistula of 0.7%. 18 Iatrogenic injury to the ureter occurred in 2% of patients. To avoid this complication, which can occur in patients with previous surgeries and distorted anatomy, preoperative planning is important, including imaging and an experienced surgical team familiar with advanced laparoscopic surgery in DIE. 26 One patient (0.67%) developed bladder atony. During the follow-up, there was complete resolution of symptoms. Marty et al. 27 described an incidence of 1.8% of bladder atony necessitating ≥ 3 weeks of daily self-catheterization. Some authors have explained that segmental rectal resection, which requires circular dissection of the rectum and wide dissection below the nodule, exposes patients to more functional morbidity than the discoid resection technique, which leads to less risk for damage to the plexus. 28

In our cohort, the conversion rate to laparotomy was very low, which is similar to other series. 29 Deep infiltrating endometriosis invading the intestine is a challenging condition, and intraoperative technical difficulty, level of laparoscopic complexity, and surgeon inexperience are some of the main risk factors for conversion to open surgery. 30 In colorectal surgery, patients who underwent conversion to open surgery had higher mortality, higher overall morbidity, longer lengths of hospitalization, and increased hospital charges. 31 Surgical recurrence, meaning a need for another bowel resection, was similar to other studies. 16 25 32 All of our patients were successfully treated with a second laparoscopy.

Our study has several limitations. First, long-term postoperative functional outcomes, such as evacuation and/or incontinence outcomes and urinary disorders, were not evaluated. However, an assessment of subjective wellbeing and intestinal and urinary symptoms was carried out and published elsewhere. 33 Secondly, although a prospective database was used, this study was limited by its retrospective nature. Third, some important data was not available in many patients, such as opening of the vagina, which is known to be a cause of postoperative complications. However, in our cohort, we did not observe any cases of rectal fistula or dehiscence.

## Conclusion

In summary, the present study confirms that laparoscopic intestinal resection for DIE has a low postoperative complication rate and a low need for temporary stoma.
